# An Interactive Method Based on the Live Wire for Segmentation of the Breast in Mammography Images

**DOI:** 10.1155/2014/954148

**Published:** 2014-06-15

**Authors:** Zhang Zewei, Wang Tianyue, Guo Li, Wang Tingting, Xu Lu

**Affiliations:** School of Medical Imaging, Tianjin Medical University, Tianjin 300203, China

## Abstract

In order to improve accuracy of computer-aided diagnosis of breast lumps, the authors introduce an improved interactive segmentation method based on Live Wire. This paper presents the Gabor filters and FCM clustering algorithm is introduced to the Live Wire cost function definition. According to the image FCM analysis for image edge enhancement, we eliminate the interference of weak edge and access external features clear segmentation results of breast lumps through improving Live Wire on two cases of breast segmentation data. Compared with the traditional method of image segmentation, experimental results show that the method achieves more accurate segmentation of breast lumps and provides more accurate objective basis on quantitative and qualitative analysis of breast lumps.

## 1. Introduction

According to the Cancer Registration Annual Report 2013 statistics [1], breast cancer has the highest morbidity and mortality in women all over the world. Over one-quarter (29%) of new cancer cases in females are attributed to breast cancer in Americans. Breast cancer is always the most common malignancy in China, and the incidence has been increasing in recent years. Therefore, early detection has an impact on prognosis. Because of the advantages of noninvasion, low cost, simplicity, and high resolution, Mammography X-ray imaging is widely used in medical clinical diagnosis.

At present, it mainly depends on the professional physician manual operation to distinguish the difference between benign and malignant breast neoplasm and make the border, which leads to heavy workload poor efficiency. With the help of computer segmentation for the breast mass, the doctor can analyze quantitatively or qualitatively the lesions in ROI.

However, because breast tissues are complex and heterogeneous, and Mammography X-ray imaging has disadvantages of low contrast, noises, and artifacts, the results are imperfect for traditional segmentation such as thresholding, region growing, watershed, and FCM.

In recent years, a large number of researches have been done in Mammography X-ray imaging to improve the quality of segmentation. Paliwal presented a new approach for preprocessing and segmenting out the infiltration and tumor regions from digital mammograms by using two techniques involving iterative and noniterative algorithms of Delaunay triangulation [2]. Kurt et al. have implemented mammography image enhancement using wavelet transform, CLAHE, and anisotropic diffusion filter then rough pectoral muscle extraction for false region reduction and better segmentation. Next they have used Rough entropy to define a threshold and then fuzzy based microcalcification enhancement, after these microcalcifications have been segmented using an iterative detection algorithm [3]. Marungo and Taylor combined thresholding with seeded region growing, which can perform rapid automated breast region segmentation with acceptable results in 98.15% of the dataset's 10,411 images [4]. Rahmati et al. presented a computer-aided approach to segmenting suspicious lesions in digital mammograms, based on a novel maximum likelihood active contour model using level sets (MLACMLS) [5]. Yuvaraj and Ragupathy proposed a new computer aided mass segmentation and classification scheme. Five statistical features were selected for the classification of mass [6]. Ullah et al. proposed a new thresholding method for segmentation that is based on DyWT, moment preserving principal, and Gamma function [7]. Sheet et al. presented a completely automatic system for detection and segmentation of breast lesions in 2D ultrasound images. They employ random forests for learning of tissue specific primal to discriminate breast lesions from surrounding normal tissues. This enables it to detect lesions of multiple shapes and sizes as well as discriminate between hypoechoic lesion from associated posterior acoustic shadowing [8]. Tunali and Kiliç presented a method for segmentation of benign and malignant masses found on mammograms. After adding low valued pixels outside of mammogram, Chan-Vese active contour algorithm with new stopping criteria was implemented [9]. Song et al. developed a new segmentation method by use of plane fitting and dynamic programming, which achieved a relatively high performance level. The new segmentation method would be useful for improving the accuracy of computerized detection and classification of breast cancer in mammography [10]. Kuo et al. evaluated a 3D lesion segmentation method, which they had previously developed for breast CT, and investigated its robustness on lesions on 3D breast ultrasound images [11]. Cordeiro et al. presented the GrowCut technique to segment tumor regions of digitized mammograms available in the minimias database [12].

There are three kinds of interactive segmentation methods [13]. One is based on closed initial contour segmentation method; active contour model and level set methods are widely used in computer vision; they have the strong capability of noise immunity, but they are sensitive to the image contrast. Therefore these methods are still not perfect for Mammography X-ray imaging. Another is based on the initial target points and background points; random walk and graph cut methods are the typical representative of this kind of medical segmentation methods. The results are affected by the location and the number of the initial target points and background points; the result has significant influence on the user experience and the repeatability is low. The third is based on the marked points on the edge; Live Wire is one of the important representatives; it has been widely implemented in medical image segmentation. Live Wire method has the less manual intervention and high-precision for the blur boundary of Mammography X-ray imaging.

**Figure 1:**
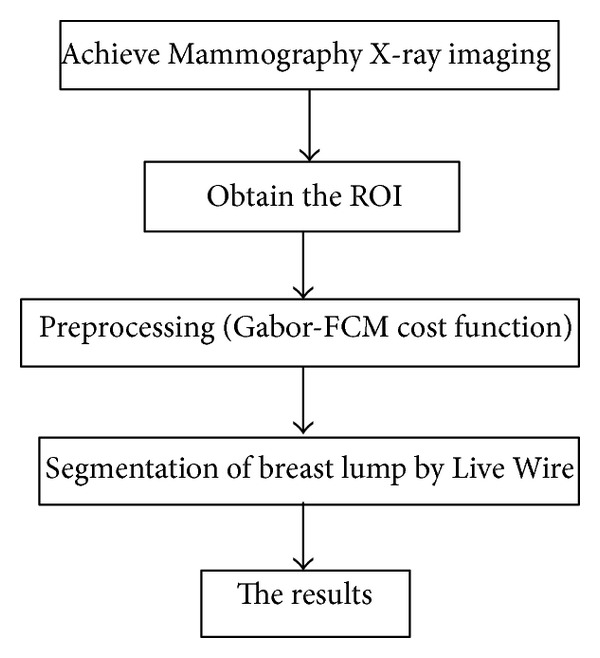
The procedure flow of the proposed algorithm.

This paper presents a semiautomatic segmentation method based on Gabor FCM algorithm for the cost function in Live Wire algorithm. In recent years, the Gabor FCM as a mathematical tool has been widely used in image segmentation. We present the Gabor filters and FCM clustering algorithm is calculated to the Live Wire cost function definition, and, according to the image FCM analysis for image edge enhancement, to eliminate the interference of weak edge, access external features clear segmentation results of breast lumps. The procedure of the proposed algorithm is shown in Figure 1.

## 2. Live Wire Algorithm

Live Wire is a famous classic interactive algorithm, first proposed by Mortensen and Barrett [14–16]. The algorithm produces reasonable cost function to construct the optimal path between the starting point and ending point provided by the user, so as to achieve the purpose of edge segmentation.

The optimal path from each pixel is determined at interactive speeds by computing an optimal spanning tree of the image using an efficient implementation of Dijkstra's graph searching algorithm. The basic idea is to formulate the image as a weighted 356 graph where pixels represent nodes with directed, weighted edges connecting each pixel with its 8 adjacent neighbors.

The key issue of Live Wire algorithms is the construction of the cost function. Theoretically, the cost function should contain all the information about the lesion edges, including gradient magnitude function, gradient direction function, and Laplace zero crossing point; the cost function of classical Live Wire algorithms is defined as follows:

(1) $$\begin{array}{c} C\left(p,q\right)=w_{Z}f_{Z}\left(q\right)+w_{G}f_{G}\left(q\right)+w_{D}f_{D}\left(p,q\right), \end{array}$$

where each *w* is the weight of the corresponding feature function. Note that, empirically, weights of *W*_*Z*_ = 0 : 43; *W*_*G*_ = 0 : 43; *W*_*D*_ = 0 : 14. *f*_*Z*_ represents formulation of Laplace zero crossing point, *f*_*G*_ represents formulation of gradient magnitude function, and *f*_*D*_ represents formulation of gradient direction function.

Laplace zero crossing point (*f*_*Z*_) is a binarization Eigen function which is used to localize edge. The Laplacian image zero-crossing corresponds to points of maximal gradient magnitude. Thus, Laplacian zero-crossing represents “good” edge properties and should have a low local cost. If *L*(*q*) is the Laplacian of an image at the pixel *q*, then

(2) $$\begin{array}{c} f_{Z}=\{\begin{array}{cc} 0, & L\left(q\right)=0\\ 1, & L\left(q\right)\neq 0, \end{array} \end{array}$$

where *L*(*q*) is the Laplacian of the original image, at a pixel *q*. If a pixel is on a zero-crossing then the component cost for all links to that pixel is low; otherwise it is high. However, gradient magnitude provides a direct correlation between edge strength and local cost. Multiscale gradient magnitude (*f*_*G*_) is as follows:

(3) $$\begin{array}{c} f_{G}\left(q\right)=1-\frac{G\left(q\right)}{\max\left(G\right)}, \end{array}$$

where *G* is the gradient magnitude of the *q* pixel. If *I*_*X*_ and *I*_*Y*_ represent the partials of an image **I** in *X* and *Y*, respectively, then the gradient magnitude is $G=\sqrt{I_{x}^{2}+I_{y}^{2}}$. *Max*⁡⁡(*G*) represents the maximum value in the image. *f*_*D*_(*p*, *q*) represents the local cost on the directed gradient from pixel *p* to the neighboring pixel *q*.

The formulation of the gradient direction feature cost is calculated as follows:

(4) $$\begin{array}{c} f_{D}\left(p,q\right)=\frac{2}{3\pi }\left\{\cos{\left[d_{p}\left(p,q\right)\right]}^{-1}+\cos{\left[d_{q}\left(p,q\right)\right]}^{-1}\right\}, \end{array}$$

where

(5) dp(p,q)=D′(p)·L(p,q),dq(p,q)=L(p,q)·D′(q),

where *D*(*p*) is a unit vector of the gradient direction at a point  *p*  and defining *D*′(*p*) as the unit vector perpendicular to *D*(*p*), which is defined as follows:

(6) $$\begin{array}{c} D^{\prime }\left(p\right)=\left(I_{y}\left(p\right),-I_{x}\left(p\right)\right), \end{array}$$

where direction of the unit vector is defined by *I*_*x*_ and *I*_*y*_, at a pixel *p*. The unit edge vector between pixels *p* and  *q*  is defined as

(7) $$\begin{array}{c} L\left(p,q\right)=\{\begin{array}{cc} q-p, & D^{\prime }\left(p\right)\cdot \left(q-p\right)\geq 0\\ p-q, & D^{\prime }\left(p\right)\cdot \left(q-p\right)<0. \end{array} \end{array}$$

## 3. Improve the Cost Function

Classical Live Wire algorithm used the Laplace zero-crossing as the cost function. Its segmentation results are sensitive to the Gauss function's parameter *δ*. Image edge becomes more blurry with the value of *δ* increasing. However, when the value of *δ* is small, the segmentation results cannot fully suppress the noise. We cannot distinguish between a strong edge and a weak edge basis of above cost function. In 2005, Yang et al. [17] proposed canny operator instead of Laplace zero-crossing in the cost function of Live Wire. The segmentation results have improved, however, the use of edge detection operator as the cost function make appear edge point error tracking, pseudoedge, discontinuous edges, and edge information loss.

Texture analysis plays an important role in variable of applications of medical image analysis for tumors segmentation based on local spatial variations of intensity. The pathological changes in lesion area result in the difference between the lesion and the surrounding normal tissue based on the texture properties in medical image. In this paper, we introduce a Gabor FCM approach to improving the Live Wire algorithm to segment the breast lumps.

### 3.1. Gabor Filter

The Gabor filter is used as an edge detector based on texture to detect the texture and gradient of the image. First, consider the Gabor filter which is defined as follows:

(8) $$\begin{array}{c} G=\exp\left(-\frac{u^{2}+\gamma v^{2}}{2\sigma^{2}}\right)\cdot \cos\left(2\pi fu+\varphi \right),\\ u=\left(x_{j}-x_{i}\right)\cdot \cos\;\theta +\left(y_{j}-y_{i}\right)\cdot \sin\;\theta ,\\ v=-\left(x_{j}-x_{i}\right)\cdot \sin\;\theta +\left(y_{j}-y_{i}\right)\cdot \cos\;\theta , \end{array}$$

where (*x*_*i*_, *y*_*i*_) is the center location of the filter, *σ* standard deviation is utilized to normalize the size of filter, and *f* is the bet space frequency bandwidth of the filter.

The selection of Gabor parameters has been a focus for the long-term research of Gabor based image processing. A large number of works have been done on this important issue. In this paper, according to the symmetry in the filter, we will choose spatial orientations (*θ*) of 0°, 30°, 60°, 90°, 120°, and 150°. The radial frequency (*f*) is often selected as follows:

(9) fH=0.25+2i−0.5N  0.25≤fH<0.5,fL=0.25−2i−0.5N  0<f<0.25,   i=1,2,…,log⁡2(N/8),

where *f*_*H*_ is higher frequencies, *f*_*L*_ is lower frequencies, and the frequency is normalized by the image size* N*, but according to the symmetry of Fourier spectrum, only half part [0 0.5] is considered in the paper.

It can be seen that the curve of the selection is much flatter in the intermediate frequency. This indicates that choice of central frequency does have finer resolutions in the intermediate frequency (around 0.25). The most of the spectral energy of natural image often centers at low frequency, so the choice of Gabor filters does not consider the very high frequency band of image.

### 3.2. Fuzzy c-Means

Fuzzy c-means (FCM) is a method of clustering which allows one piece of data to belong to two or more clusters. This algorithm works by assigning membership to each data point corresponding to each cluster center on the basis of distance between the cluster center and the data point. The more the data is near to the cluster center, the more is its membership towards the particular cluster center. It is based on minimization of the following objective function:

(10) $$\begin{array}{c} J_{\text{FCM}}=\overset{c}{\underset{i=1}{\sum }}\;\overset{n}{\underset{k=1}{\sum }}\mu_{ik}^{m}{||x_{k}-v_{i}||}^{2}, \end{array}$$

where *v*_*i*_ ∈ *V*, *V* = (*v*_1_, *v*_2_,…, *v*_*c*_) ∈ *R* ^*c*×*p*^ is the cluster center of* c*; *U* = {*μ*_*ik*_} ∈ *R* ^*c*×*n*^ (*μ*_*ik*_ ∈ [0,1],  ∑_*i*=1_^*c*^ *μ*_*ik*_ = 1) is the membership matrix; *m* ∈ (1, *∞*) is the weighting exponent, as usual *m* = 2. Membership matrix *U* and cluster centroids *V* can be obtained after minimization of the object function. Membership matrix *U* and cluster centroids *V* are calculated as follows:

(11) μik=(∑j=1c(||vi−xk||||vj−xk||)2/(m−1)),−1vi=∑k=1nμikmxk∑k=1nμikm.

The condition of termination is as follows:

(12) $$\begin{array}{c} \left|J_{t}-J_{t-1}\right|<\varepsilon , \end{array}$$

where *J*_*t*_ and *J*_*t*−1_ are the current objective function and last objective function, *ε* > 0 is a small number which our choice is *ε* = 10^−5^. In our experiment, we found that ten clusters are sufficient to separate the breast lumps centers, the fuzzy boundary, and others clearly.

## 4. Experimental Results and the Analysis

### 4.1. Experimental Data

In order to verify the effectiveness of the algorithm, we applied the algorithm on two parts of experimental data; the first part of data includes 16 cases which are provided by* Tianjin Medical University General Hospital Radiology Department*. The images are clear and voxels of each image are isotropous, which ensure the accuracy of the volume segmentation. The second part of the data consists of 68 cases which are extracted from a public database of Digital Database for Screening Mammography (DDSM), provided by the* U.S. Army Medical Research and Materiel Command Breast Cancer Research Program.* Four radiological diagnosticians on breast manually outlined the lumps on these data and obtained the diameter and the volume of the lumps.

### 4.2. The Segmentation Results

The results of our Gabor FCM Live Wire algorithm used in this paper proved that Gabor FCM fits better than traditional Laplace zero crossing point as cost function of Live Wire algorithm.

**Figure 2:**
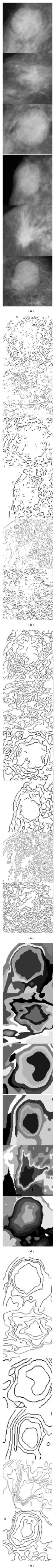
Preprocess for the improvement of the cost function of Live Wire.

Figure 2(a) is the original image of breast X-ray Mammography. In this paper, we captured the region of interest of breast lumps from each image to reduce the number of unnecessary pixels for segregating out the lumps, which could improve the segmentation accuracy of the algorithm and the computational efficiency.

Figure 2(b) is the edge curves obtained by using traditional Live Wire algorithm. Laplace zero-crossing point is not good at edge extraction of breast lumps with complex and changeable organizational structure, strong noise, and low contrast, which make the curves extracted by Laplace zero-crossing point discontinuous and complex and have a lot of false contour. Thus, traditional Live Wire is susceptible to the false contour.

Figure 2(c) shows the results of edge extraction for each lump image by applying Canny edge operator, and there are many edge curves in the results. Particularly, the second and third cases have invasive breast cancer which was characterized by irregular shape and dim edge and surrounded by funicular, tufted, or trabecular structures and their segmentation results were discursive. The detected multiple edge curves of the lumps using canny edge operator have great interference in extracting the accurate edge curves to accurately locate the target boundary.

Figure 2(d) is the processed result of Gabor FCM. We can see that there are several gray value layers from the center of the breast lumps to the outside, which can accurately reflect the general shape and size of the lump, as well as the layers and area of the halo around the lump. Thus it provides convenience for evaluating clinical breast tumor at different respects.

Figure 2(e) shows the obtained edge curves extracted from Gabor FCM processed images. These edge curves were regularly closed around the breast lump region. The curves replaced the results of using Laplace zero-crossing point and were used as the improved cost function in Live Wire algorithm, by which we acquired the target contour which is closer to the essence of tumor center or the halo around the tumor and improved the accuracy of the segmentation.

**Figure 3:**
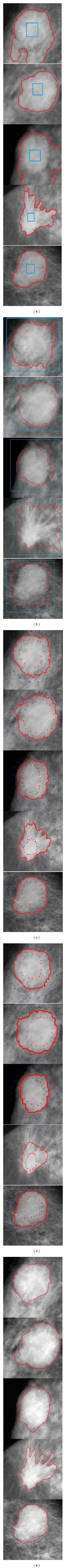
The comparison with other interactive segmentation methods.

Figure 3(a) is the segmentation results of Snake algorithm.  Figure 3(b) is the segmentation results of Level Set algorithm. Both of the algorithms mistakenly segregated part of the normal breast tissue into the diseased tissue and could not converge to the real edges.  Figure 3(c) is the segmentation results of Graph cut algorithm.  Figure 3(d) is the segmentation results of Random Walk algorithm. Although the segmentation results are ideal, it requires a lot of manual operations. It needs 20 target points and 20 background points to get ideal results. In the fourth row, we can see that it failed to converge to the correct position at the edge of concave which has a large curvature.  Figure 3(e) is the segmentation results of our improved algorithm in this paper.

The normal tissues which were missegmented in Figures 3(a) and 3(b) were segmented correctly. Using the improved algorithm in this paper, it only needs to mark 10 points on the edge of lump, which can greatly reduce the manual operations and the impact of operational personnel experience on segmentation results and improve the accuracy and efficiency of segmentation.

**Figure 4:**
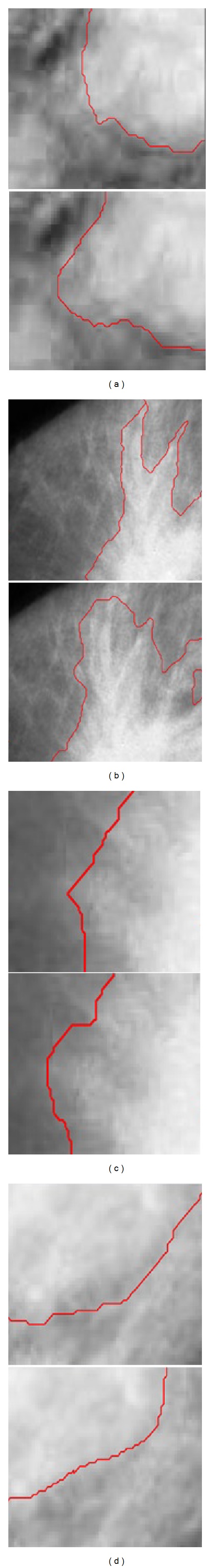
The comparison between the traditional Live Wire and the improved Live Wire.

The top row in Figure 4 is the segmentation results of traditional Live Wire algorithm; the second row is the segmentation results of the improved algorithm in this paper. It can be seen that the segmentation results of the breast lump edges obtained by traditional Live Wire algorithm are not ideal and are insensitive to fuzzy halo around the breast lump. From the segmentation results of traditional Live Wire algorithm in Figure 4(a), we can see that the obtained contour is too restrained to extract the whole focus areas, and part of the lesions areas are divided into normal tissues by mistake, which may have bad effect on clinical diagnosis.

Figure 4(b) shows that traditional Live Wire algorithm can only segment out the substantial part of the cancerous tissue and fail to classify the fuzzy halo around the substantial part of the cancerous tissue into focus lesions areas, which may result in omission of lesions tissue and reducing the algorithm value of assisted diagnose breast cancer. In Figure 4(c), inconsistent with the smooth edge of breast lumps, the contour obtained from traditional Live Wire algorithm showed break angles, which may be related to the false contour and discontinuous curves due to the shortcoming of Laplace zero-crossing point in detecting the edge of breast lumps which have complex and changeable organizational structure, strong noise, and low contrast. In Figure 4(d), we can see that the edge of the lesion area obtained by using traditional Live Wire algorithm does not converge completely and thus diverges outwards excessively. The segmentation results contain some normal tissue with low density, which may exaggerate the patient's condition in clinical diagnosis. From the four figures we can see that the segmentation results obtained by using improved algorithm in this study are more fitting to the edges of the lesion areas and highly sensitive to the lesion area having weak edges. The generated target contours are smooth and accurate, which can meet the demands of clinical diagnosis and improve the value of the computer-aided diagnosis of the breast cancer.

**Figure 5:**
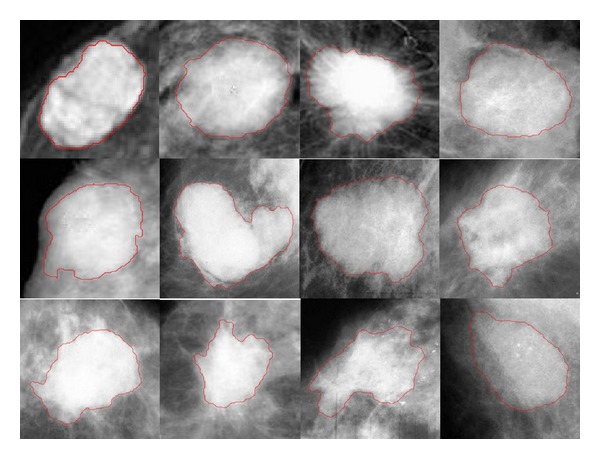
The segmentation results of the improved Live Wire algorithm.

Figure 5 shows the image segmentation results of a set of breast cases by using the improved Live Wire algorithm in this paper. From the images of breast cases, we can see that the lump shape of most of the breast cases is irregular and the boundaries are fuzzy, which cannot meet the request of image segmentation for automatic segmentation methods. By using the Live Wire interactive segmentation method in this paper, we quickly obtained the smooth and accurate lump edges according to the gray information of the images. So the improved Live Wire interactive segmentation method can reduce the doctor's interactive operation and provide assistance to the doctors to analyze the breast cases.

**Figure 6:**
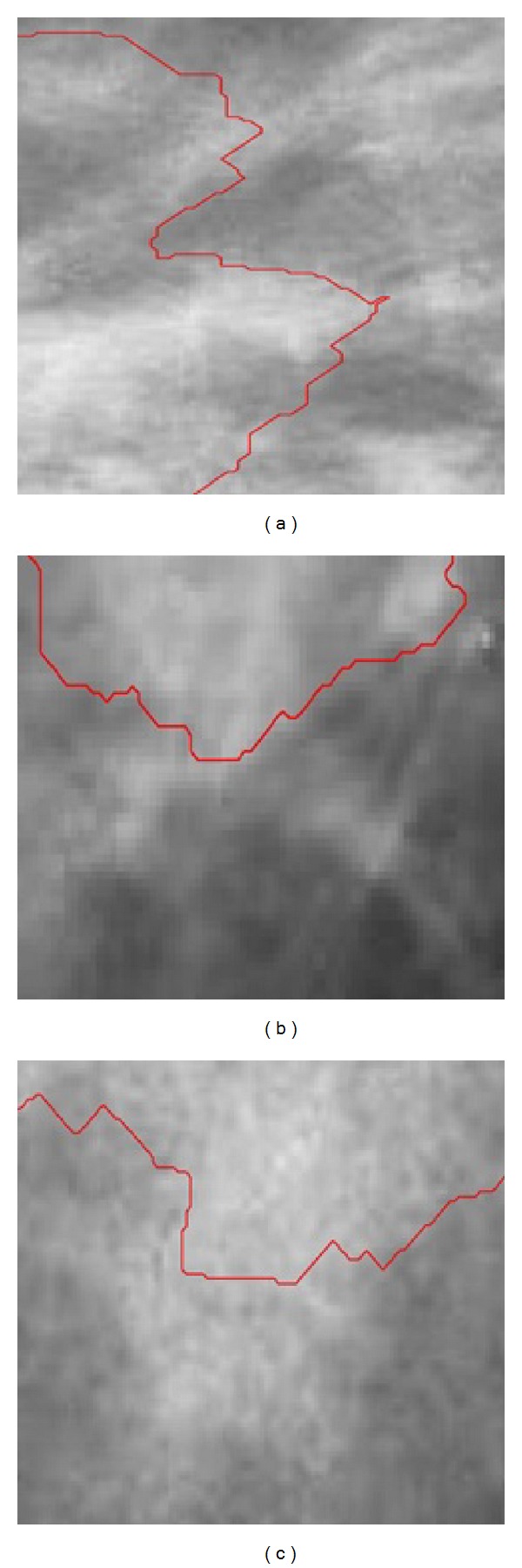
Examples of segmentation failures for breast lump.

These are the examples of segmentation failures for breast lump. The failures were due to a removal of a part of the lumps (Figure 6). It is more difficult to segment breast lumps due to the large variations in the intensities and vague boundaries, such as Figure 6(a).  Figure 6(b) shows the segmentation of breast lump. For this case, different from the previous, a large wrong result of Live Wire occurs, making lumps identification more difficult. For this breast lump in Figure 6(c), a part of the lumps was missed in the final segmentation. Through Figure 6 we can obtain the reason which led to failure in the detection of this nonhomogeneity lumps. Their inhomogeneous density distribution made it hard to obtain an exact edge as possible.

### 4.3. Assessment of Segmentation Performance

In order to quantitatively evaluate the accuracy of the segmentation results, we have carried out several sets of experiments to compare the computer automatic segmentation results with the standard lesion edge manually outlined by clinical physicians at the pixel level. According to the clinical testing criterion, the accuracy in terms of true positive ratio (TP), false positive ratio (FP), false negative ratio (FN), and misclassification error (ME) was used as quantitative indices, which are defined as follows:

(13) $$\begin{array}{c} \text{TP}=\frac{\text{Area}\left\{S_{A}\cap S_{m}\right\}}{\text{Area}\left\{S_{B}\right\}},\\ \text{FP}=\frac{\text{Area}\left\{S_{A}\cup S_{B}-S_{B}\right\}}{\text{Area}\left\{S_{B}\right\}},\\ \text{FN}=\frac{\text{Area}\left\{S_{A}\cup S_{B}-S_{A}\right\}}{\text{Area}\left\{S_{B}\right\}},\\ \text{ME}=\frac{\text{Area}\left\{S_{A}\cup S_{B}\right\}-\text{Area}\left\{S_{A}\cap S_{B}\right\}}{\text{Area}\left\{S_{A}\cup S_{B}\right\}}, \end{array}$$

where *S*_*A*_ is the area of computer automatic segmentation and *S*_*B*_ is the area of manually describe.

When TP is high, it indicates that the segmentation result will be less missed lesions, with high-value of clinical diagnosis. When FP is high, it indicates that the segmentation results diverge outwardly, some of the lesions classified as normal tissue region; the area of segmentation results is larger than the area of actual lesion. This causes physician to assess disease excessively, excise part of normal tissue in the operation, and give patients greater body injury. When FN is high, it indicates that the segmentation results in excessive convergence, which causes the physician to ignore some lesions, and thus affects clinical diagnosis. The smaller the ME value, the more ideal the segmentation result. The segmentation results are more consistent with the actual lesion. When ME = 0, the computer automatic segmentation results are fully consistent with the manual segmentation results.

The comparison between our algorithm and other algorithms in quantitative indices is represented in Table 1. It can be clearly seen that the average TP (96.64%) of our algorithm exceeded that of other algorithms, and the average FN (4.07%) of our algorithm is much less than that of other algorithms. It manifests that our algorithm can recognize the lesion accurately and misses lesions rarely. However, as far as the average FP is concerned, it can be seen that our algorithm is slightly worse than the random walk algorithm, but the average FN (4.07%) of our algorithm is far less than that (23.48%) of the random walk algorithm. The results of experiment show that the random walk algorithm is not sensitive to the weak edges surrounding the breast lumps. The segmentation results show excessive convergence, which results in ignoring some lesions and missing some pathological tissue during the procedure of operation.

Data in Table 2 demonstrates the comparison between our algorithm and the classical Live Wire algorithm in quantitative indices. It can be seen that our algorithm is more satisfactory than the classical Live Wire algorithm in all quantitative indices. From the observation of average ME, our algorithm is superior to the classical Live Wire algorithm, which indicates that the segmentation result is more consistent with the actual lesion and can provide accurate reference for clinical physicians.

## 5. Conclusions

The edge information of breast mass lumps which was obtained from computer automatic segmentation method provides important data for the diagnosis of breast cancer. Owing to edge of breast lumps, which is very fuzzy, much noise and low contrast, the classical Live Wire algorithm and some other widely used algorithms are not able to accurately identify the edge of breast lumps. This paper presents an improved Live Wire interactive segmentation method which uses Gabor-FCM as the cost function, through the interactive segmentation to extract the edge of breast lumps. Based on a lot of experiments we can arrive at the conclusion that our algorithm can accurately extract the boundary of the lesions, as well as its capability of location, detection and segmentation has more significant improvement than the classical Live Wire algorithm. Our algorithm sufficiently satisfies the requirements of clinical diagnosis and comes up to the auxiliary diagnostic standard. Moreover, our algorithm provides accurate and reliable data for the physicians. So the method used by this paper will be widely applied to the clinical diagnosis.

## Figures and Tables

**Table 1:** Comparison between our algorithms and other algorithms in quantitative indices.

Method	TP (%)	FP (%)	FN (%)	ME (%)
Our algorithm	96.64	1.97	4.07	5.91
Level set	92.76	35.17	7.61	29.50
Graph cut	89.65	3.65	10.86	13.95
Random walk	77.46	1.33	23.48	24.56
Snake	94.85	36.86	6.22	26.95

**Table 2:** Comparison between our algorithm and the classical Live Wire algorithm.

Method	TP (%)	FP (%)	FN (%)	ME (%)
Our algorithm	96.64	1.97	4.07	5.91
Live Wire	92.07	4.55	8.65	12.42
